# 2-[(*E*)-2-Hy­droxy-3-methoxy­benzyl­idene]-*N*-methyl­hydrazinecarbothio­amide

**DOI:** 10.1107/S1600536812049847

**Published:** 2012-12-08

**Authors:** B. S. Shankara, N. Shashidhar, Yogesh Prakash Patil, P. Murali Krishna, Munirathinam Nethaji

**Affiliations:** a Department of Chemistry, Sri Krishna Institute of Technology, Bangalore 560 090, India; b Department of Chemistry, S. D. M. College of Engineering and Technology, Dharwad 580 002, India; c Department of Inorganic and Physical Chemistry, Indian Institute of Science, Bangalore 560 012, India; d Department of Chemistry, M. S. Ramaiah Institute of Technology, Bangalore 560 054, Karnataka, India

## Abstract

In the crystal structure of the title compound, C_11_H_15_N_3_O_2_S, mol­ecules are linked by pairs of N—H⋯O and O—H⋯S hydrogen, forming inversion dimers. These dimers are linked by N—H⋯S hydrogen bonds, forming double-stranded chains propagating along the *b*-axis direction. The two C atoms of the end chain of the mol­ecule are disordered over two sets os sites [occupancy ratio 0.574 (9):0.426 (9)].

## Related literature

For related structures, see: Joseph *et al.* (2006 ▶); Ren-Gao Zhao *et al.*(2008 ▶). For the biological activity of thio­semicarbazone Schiff bases, see: Kasuga *et al.* (2003 ▶); Murali *et al.* (2008 ▶, 2009 ▶); Paterson & Donnelly (2011 ▶).
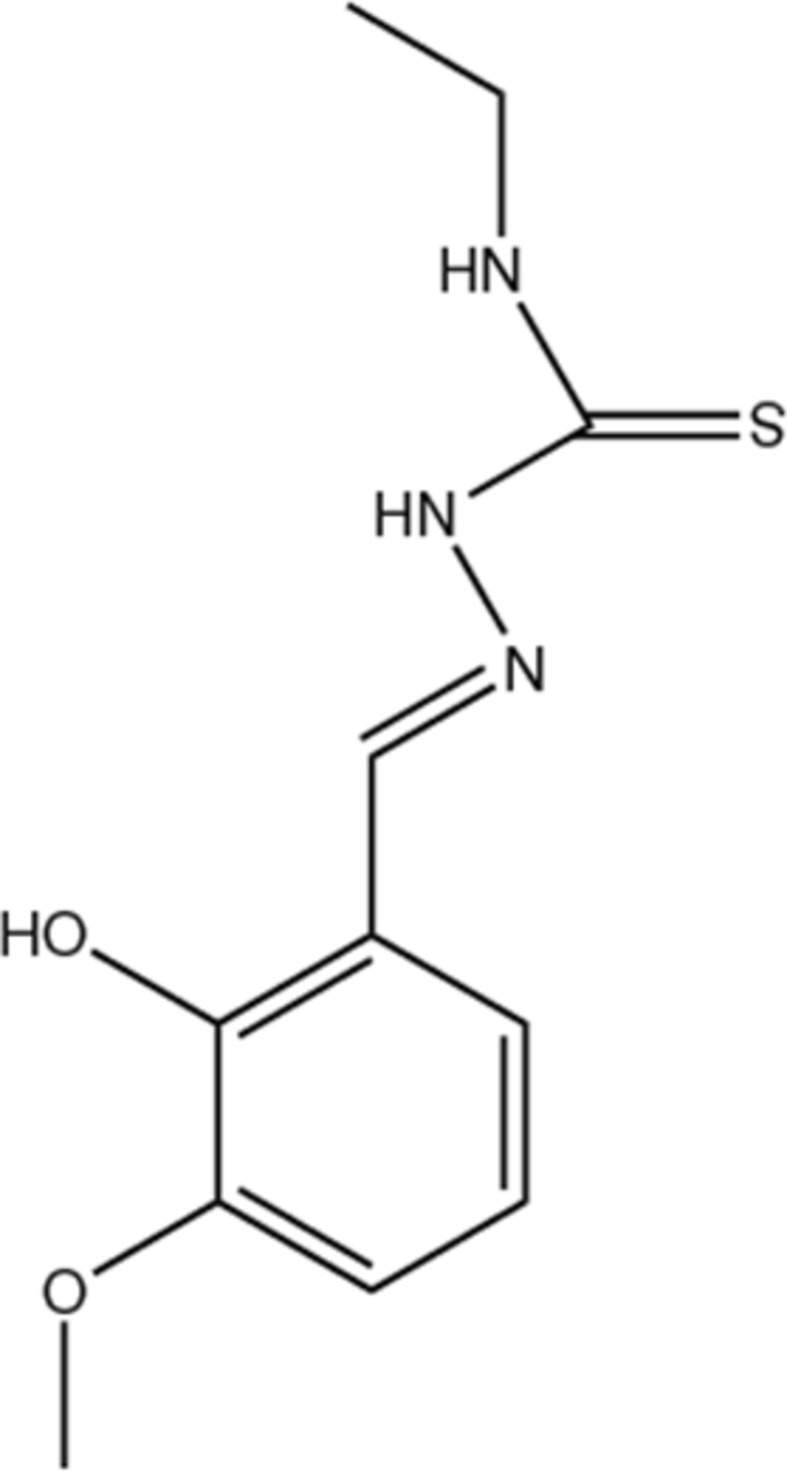


## Experimental

### 

#### Crystal data

C_11_H_15_N_3_O_2_S
*M* *_r_* = 253.32
Monoclinic, 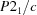

*a* = 13.251 (6) Å
*b* = 6.185 (3) Å
*c* = 16.380 (8) Å
β = 113.153 (7)°
*V* = 1234.4 (11) Å^3^
*Z* = 4
Mo *K*α radiation
μ = 0.26 mm^−1^
*T* = 293 K
0.26 × 0.09 × 0.05 mm

#### Data collection

Bruker APEXII CCD diffractometer
Absorption correction: multi-scan (*SADABS*; Bruker, 2006 ▶) *T*_min_ = 0.936, *T*_max_ = 0.987
7373 measured reflections
2433 independent reflections
1666 reflections with *I* > 2σ(*I*)
*R*_int_ = 0.030

#### Refinement

*R*[*F* ^2^ > 2σ(*F* ^2^)] = 0.064
*wR*(*F* ^2^) = 0.180
*S* = 1.06
2433 reflections
175 parameters
4 restraints
H atoms treated by a mixture of independent and constrained refinement
Δρ_max_ = 0.46 e Å^−3^
Δρ_min_ = −0.37 e Å^−3^

### 

Data collection: *APEX2* (Bruker, 2006 ▶); cell refinement: *SAINT* (Bruker, 2006 ▶); data reduction: *SAINT*; program(s) used to solve structure: *SHELXS97* (Sheldrick, 2008 ▶); program(s) used to refine structure: *SHELXL97* (Sheldrick, 2008 ▶); molecular graphics: *ORTEP-3* (Farrugia, 2012 ▶); software used to prepare material for publication: *WinGX* (Farrugia, 2012 ▶).

## Supplementary Material

Crystal structure: contains datablock(s) I, global. DOI: 10.1107/S1600536812049847/gw2126sup1.cif

Structure factors: contains datablock(s) I. DOI: 10.1107/S1600536812049847/gw2126Isup2.hkl

Supplementary material file. DOI: 10.1107/S1600536812049847/gw2126Isup3.cml

Additional supplementary materials:  crystallographic information; 3D view; checkCIF report

## Figures and Tables

**Table 1:** Hydrogen-bond geometry (Å, °)

*D*—H⋯*A*	*D*—H	H⋯*A*	*D*⋯*A*	*D*—H⋯*A*
O1—H1⋯S1^i^	0.87 (4)	2.42 (4)	3.169 (3)	145 (3)
N2—H2⋯O1^i^	0.82 (3)	2.29 (4)	3.010 (4)	147 (3)

Symmetry code: (i) 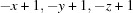.

**Fig. 1.**
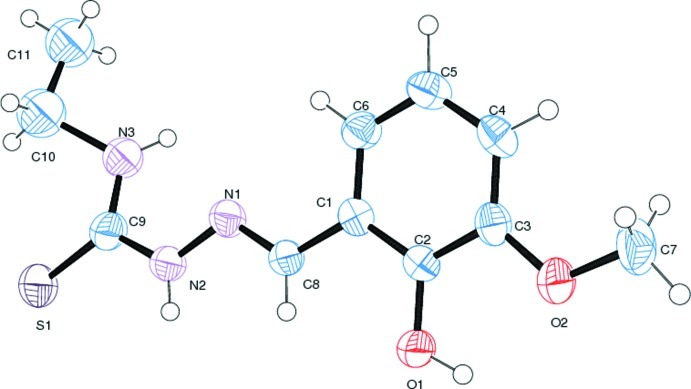
The structure of title the compound (I)

**Fig. 2.**
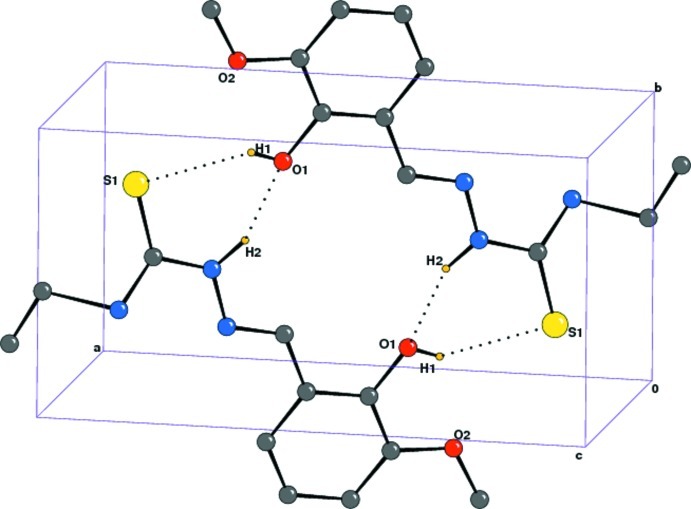
Packing diagram of (I)

## supplementary crystallographic information

### Comment

Thiosemicarbazones emerged an important class of sulfur and nitrogen containing Schiff-bases due to their chemistry and potentially beneficial biological activities, such as antitumor,antibacterial, antiviral and antimalarial activities (Kasuga *et al.*, 2003; Paterson & Donnelly, 2011). In a continuation of our studies on thiosemicarbazone Schiff-bases, we report the synthesis and crystal structure of the title compound, (I).

In (I) (Fig. 1), all bond lengths and angles are normal and in a good agreement with those found in the literature (Joseph *et al.*, 2006).There is one molecule in the assymetric unit with the end atoms thermally disordered. This molecule exhibits intermolecular N—H···O and O—H···S hydrogen bonds (Table 2) forming a dimer along *b* axis. These dimers give rise to a zigzag pattern seen along *c* axis. Further the molecules are packed by weak π···π interactions [centroid–centroid distance 4.495 (5) Å]

### Experimental

The title compound (I)was synthesized by the reaction of 2-hydroxy-3-methoxy benzaldehyde (10 g, 0.1 mol) in 250 ml round bottom flask, 5% acetic acid-water solution of 4 N-methyl hydrazinecarbothioamide (0.1 mol) in ethanol solution and refluxed on a steam bath for 30–45 minutes. The crystalline product which formed was collected by filtration, washed several times with hot water and, then ether, finally dried in vacuo. Then good quality crystals (I)were obtained in a 1:1 mixture of ethanol and n-hexane.

### Refinement

The hydrogen atoms were located with the help of difference fourier maps. Hydrogen atoms for C7, C6 were positioned geometrically and refined using a riding model.

The end group of N-Et was disordered and modelled with the help of part command. The major component *i.e.* N3–C10–C11 is depicted in the *ORTEP* diagram. since this group is diordered over two positions, isotropic refinement is done for these 3 atoms. SADI and *DFIX* commands were used to model the disordered atoms. The hydrogen atoms were fixed for these atoms.

There are two reflections missing from the fcf file according to check cif which may be at high angle beyond the limiting sphere and not possible for recording despite the fact the data was recollected with another crystal.

### Crystal data

**Table:**

C_11_H_15_N_3_O_2_S	*F*(000) = 536
*M**_r_* = 253.32	*D*_x_ = 1.363 Mg m^−^^3^
Monoclinic, *P*2_1_/*c*	Mo *K*α radiation, λ = 0.71073 Å
Hall symbol: -P 2ybc	Cell parameters from 1499 reflections
*a* = 13.251 (6) Å	θ = 2.6–25.6°
*b* = 6.185 (3) Å	µ = 0.26 mm^−^^1^
*c* = 16.380 (8) Å	*T* = 293 K
β = 113.153 (7)°	Rectangular plate like, yellow
*V* = 1234.4 (11) Å^3^	0.26 × 0.09 × 0.05 mm
*Z* = 4	

### Data collection

**Table:**

Bruker APEXII CCD diffractometer	2433 independent reflections
Radiation source: fine-focus sealed tube	1666 reflections with *I* > 2σ(*I*)
Graphite monochromator	*R*_int_ = 0.030
Detector resolution: 0.3 pixels mm^-1^	θ_max_ = 26.0°, θ_min_ = 1.7°
φ and ω scans	*h* = −16→16
Absorption correction: multi-scan (*SADABS*; Bruker, 2006)	*k* = −6→7
*T*_min_ = 0.936, *T*_max_ = 0.987	*l* = −20→20
7373 measured reflections	

### Refinement

**Table:**

Refinement on *F*^2^	Primary atom site location: structure-invariant direct methods
Least-squares matrix: full	Secondary atom site location: difference Fourier map
*R*[*F*^2^ > 2σ(*F*^2^)] = 0.064	Hydrogen site location: inferred from neighbouring sites
*wR*(*F*^2^) = 0.180	H atoms treated by a mixture of independent and constrained refinement
*S* = 1.06	*w* = 1/[σ^2^(*F*_o_^2^) + (0.0756*P*)^2^ + 1.2001*P*] where *P* = (*F*_o_^2^ + 2*F*_c_^2^)/3
2433 reflections	(Δ/σ)_max_ < 0.001
175 parameters	Δρ_max_ = 0.46 e Å^−^^3^
4 restraints	Δρ_min_ = −0.37 e Å^−^^3^

### Special details

**Table:**

Geometry. All s.u.'s (except the s.u. in the dihedral angle between two l.s. planes) are estimated using the full covariance matrix. The cell s.u.'s are taken into account individually in the estimation of s.u.'s in distances, angles and torsion angles; correlations between s.u.'s in cell parameters are only used when they are defined by crystal symmetry. An approximate (isotropic) treatment of cell s.u.'s is used for estimating s.u.'s involving l.s. planes.
Refinement. Refinement of *F*^2^ against ALL reflections. The weighted *R*-factor *wR* and goodness of fit *S* are based on *F*^2^, conventional *R*-factors *R* are based on *F*, with *F* set to zero for negative *F*^2^. The threshold expression of *F*^2^ > 2σ(*F*^2^) is used only for calculating *R*-factors(gt) *etc*. and is not relevant to the choice of reflections for refinement. *R*-factors based on *F*^2^ are statistically about twice as large as those based on *F*, and *R*- factors based on ALL data will be even larger.

### Fractional atomic coordinates and isotropic or equivalent isotropic displacement parameters (Å^2^)

**Table:**

	*x*	*y*	*z*	*U*_iso_*/*U*_eq_	Occ. (<1)
S1	0.87523 (8)	0.71851 (17)	0.56230 (8)	0.0642 (4)	
O1	0.37812 (19)	0.2015 (4)	0.55013 (16)	0.0483 (6)	
O2	0.28859 (18)	−0.1250 (4)	0.60039 (16)	0.0515 (7)	
N1	0.7027 (2)	0.2534 (4)	0.60644 (18)	0.0419 (7)	
N2	0.7343 (2)	0.4427 (5)	0.5791 (2)	0.0462 (7)	
C1	0.5554 (2)	0.0466 (5)	0.61505 (19)	0.0355 (7)	
C2	0.4438 (2)	0.0358 (5)	0.59547 (19)	0.0356 (7)	
C3	0.3996 (3)	−0.1393 (5)	0.6244 (2)	0.0393 (7)	
C4	0.4666 (3)	−0.3038 (6)	0.6715 (2)	0.0457 (8)	
C5	0.5792 (3)	−0.2945 (6)	0.6909 (2)	0.0473 (8)	
C6	0.6224 (3)	−0.1240 (5)	0.6625 (2)	0.0420 (8)	
H6	0.6972	−0.1211	0.6748	0.050*	
C7	0.2363 (3)	−0.2905 (7)	0.6292 (3)	0.0617 (11)	
H7A	0.2530	−0.4284	0.6107	0.093*	
H7B	0.1583	−0.2680	0.6036	0.093*	
H7C	0.2621	−0.2871	0.6927	0.093*	
C8	0.5999 (3)	0.2336 (5)	0.5868 (2)	0.0386 (7)	
C9	0.8392 (3)	0.4794 (6)	0.5909 (2)	0.0491 (9)	
N3	0.9010 (4)	0.2968 (11)	0.6089 (5)	0.0524 (17)*	0.574 (9)
H3	0.8756	0.1678	0.5955	0.063*	0.574 (9)
C10	1.0329 (6)	0.3596 (12)	0.6605 (6)	0.076 (3)*	0.574 (9)
H10A	1.0552	0.4659	0.6273	0.092*	0.574 (9)
H10B	1.0491	0.4138	0.7199	0.092*	0.574 (9)
C11	1.0867 (7)	0.1453 (13)	0.6627 (7)	0.079 (3)*	0.574 (9)
H11A	1.0750	0.0528	0.7052	0.119*	0.574 (9)
H11B	1.1641	0.1661	0.6793	0.119*	0.574 (9)
H11C	1.0556	0.0797	0.6050	0.119*	0.574 (9)
N3A	0.9128 (5)	0.3405 (12)	0.6420 (6)	0.043 (2)*	0.426 (9)
H3A	0.9109	0.2887	0.6901	0.051*	0.426 (9)
C10A	1.0095 (7)	0.2744 (17)	0.6053 (6)	0.065 (3)*	0.426 (9)
H10C	0.9888	0.1537	0.5641	0.078*	0.426 (9)
H10D	1.0334	0.3959	0.5799	0.078*	0.426 (9)
C11A	1.0909 (11)	0.213 (3)	0.6964 (8)	0.111 (5)*	0.426 (9)
H11D	1.0844	0.3111	0.7395	0.167*	0.426 (9)
H11E	1.1639	0.2207	0.6977	0.167*	0.426 (9)
H11F	1.0763	0.0684	0.7100	0.167*	0.426 (9)
H1	0.309 (3)	0.176 (6)	0.535 (2)	0.055 (11)*	
H2	0.687 (3)	0.534 (6)	0.556 (2)	0.048 (11)*	
H4	0.435 (3)	−0.420 (6)	0.688 (2)	0.050 (10)*	
H5	0.634 (3)	−0.414 (6)	0.726 (2)	0.060 (10)*	
H8	0.553 (3)	0.336 (5)	0.557 (2)	0.040 (9)*	

### Atomic displacement parameters (Å^2^)

**Table:**

	*U*^11^	*U*^22^	*U*^33^	*U*^12^	*U*^13^	*U*^23^
S1	0.0450 (6)	0.0550 (6)	0.0933 (8)	−0.0108 (4)	0.0280 (5)	0.0119 (5)
O1	0.0348 (13)	0.0452 (14)	0.0638 (16)	0.0000 (11)	0.0181 (12)	0.0143 (12)
O2	0.0413 (13)	0.0519 (15)	0.0639 (15)	−0.0080 (11)	0.0236 (12)	0.0098 (12)
N1	0.0391 (15)	0.0401 (15)	0.0505 (16)	−0.0012 (12)	0.0217 (13)	0.0044 (13)
N2	0.0349 (15)	0.0393 (16)	0.066 (2)	0.0004 (13)	0.0212 (14)	0.0096 (15)
C1	0.0404 (17)	0.0351 (17)	0.0342 (16)	−0.0023 (13)	0.0181 (14)	−0.0020 (13)
C2	0.0396 (17)	0.0325 (16)	0.0359 (16)	0.0003 (13)	0.0160 (14)	0.0013 (13)
C3	0.0423 (18)	0.0400 (18)	0.0408 (17)	−0.0068 (15)	0.0218 (15)	−0.0035 (15)
C4	0.057 (2)	0.0352 (18)	0.051 (2)	−0.0042 (16)	0.0276 (18)	0.0058 (15)
C5	0.053 (2)	0.0410 (19)	0.051 (2)	0.0068 (16)	0.0245 (17)	0.0094 (16)
C6	0.0388 (17)	0.0431 (18)	0.0468 (19)	0.0040 (15)	0.0197 (15)	0.0012 (15)
C7	0.055 (2)	0.063 (3)	0.075 (3)	−0.0192 (19)	0.035 (2)	0.004 (2)
C8	0.0355 (17)	0.0373 (17)	0.0426 (18)	−0.0004 (15)	0.0151 (14)	0.0042 (15)
C9	0.0367 (18)	0.051 (2)	0.059 (2)	−0.0024 (16)	0.0194 (16)	0.0060 (17)

### Geometric parameters (Å, º)

**Table:**

S1—C9	1.676 (4)	C7—H7C	0.9600
O1—C2	1.360 (4)	C8—H8	0.89 (3)
O1—H1	0.87 (4)	C9—N3A	1.322 (8)
O2—C3	1.367 (4)	C9—N3	1.358 (7)
O2—C7	1.416 (4)	N3—C10	1.661 (8)
N1—C8	1.277 (4)	N3—H3	0.8600
N1—N2	1.376 (4)	C10—C11	1.499 (7)
N2—C9	1.346 (4)	C10—H10A	0.9700
N2—H2	0.82 (3)	C10—H10B	0.9700
C1—C2	1.387 (4)	C11—H11A	0.9600
C1—C6	1.401 (4)	C11—H11B	0.9600
C1—C8	1.454 (4)	C11—H11C	0.9600
C2—C3	1.399 (4)	N3A—C10A	1.667 (8)
C3—C4	1.371 (5)	N3A—H3A	0.8600
C4—C5	1.399 (5)	C10A—C11A	1.506 (7)
C4—H4	0.93 (4)	C10A—H10C	0.9700
C5—C6	1.366 (5)	C10A—H10D	0.9700
C5—H5	1.04 (4)	C11A—H11D	0.9600
C6—H6	0.9300	C11A—H11E	0.9600
C7—H7A	0.9600	C11A—H11F	0.9600
C7—H7B	0.9600		
			
C2—O1—H1	113 (3)	N1—C8—H8	121 (2)
C3—O2—C7	118.0 (3)	C1—C8—H8	118 (2)
C8—N1—N2	115.5 (3)	N3A—C9—N2	116.4 (4)
C9—N2—N1	121.7 (3)	N2—C9—N3	113.1 (4)
C9—N2—H2	120 (2)	N3A—C9—S1	122.0 (4)
N1—N2—H2	118 (2)	N2—C9—S1	120.0 (3)
C2—C1—C6	118.5 (3)	N3—C9—S1	125.5 (3)
C2—C1—C8	119.6 (3)	C9—N3—C10	109.9 (5)
C6—C1—C8	121.9 (3)	C9—N3—H3	125.1
O1—C2—C1	119.0 (3)	C10—N3—H3	125.1
O1—C2—C3	120.3 (3)	C11—C10—N3	101.6 (6)
C1—C2—C3	120.6 (3)	C11—C10—H10A	111.4
O2—C3—C4	126.5 (3)	N3—C10—H10A	111.4
O2—C3—C2	113.5 (3)	C11—C10—H10B	111.4
C4—C3—C2	120.0 (3)	N3—C10—H10B	111.4
C3—C4—C5	119.6 (3)	H10A—C10—H10B	109.3
C3—C4—H4	118 (2)	C9—N3A—C10A	114.2 (6)
C5—C4—H4	122 (2)	C9—N3A—H3A	122.9
C6—C5—C4	120.4 (3)	C10A—N3A—H3A	122.9
C6—C5—H5	116 (2)	C11A—C10A—N3A	93.3 (9)
C4—C5—H5	123 (2)	C11A—C10A—H10C	113.1
C5—C6—C1	120.7 (3)	N3A—C10A—H10C	113.1
C5—C6—H6	119.6	C11A—C10A—H10D	113.1
C1—C6—H6	119.6	N3A—C10A—H10D	113.1
O2—C7—H7A	109.5	H10C—C10A—H10D	110.4
O2—C7—H7B	109.5	C10A—C11A—H11D	109.5
H7A—C7—H7B	109.5	C10A—C11A—H11E	109.5
O2—C7—H7C	109.5	H11D—C11A—H11E	109.5
H7A—C7—H7C	109.5	C10A—C11A—H11F	109.5
H7B—C7—H7C	109.5	H11D—C11A—H11F	109.5
N1—C8—C1	121.5 (3)	H11E—C11A—H11F	109.5
			
C8—N1—N2—C9	−176.1 (3)	C8—C1—C6—C5	177.8 (3)
C6—C1—C2—O1	179.4 (3)	N2—N1—C8—C1	−177.1 (3)
C8—C1—C2—O1	−0.1 (4)	C2—C1—C8—N1	177.3 (3)
C6—C1—C2—C3	1.5 (4)	C6—C1—C8—N1	−2.2 (5)
C8—C1—C2—C3	−178.0 (3)	N1—N2—C9—N3A	−10.2 (6)
C7—O2—C3—C4	2.6 (5)	N1—N2—C9—N3	16.6 (6)
C7—O2—C3—C2	−177.8 (3)	N1—N2—C9—S1	−176.2 (3)
O1—C2—C3—O2	1.6 (4)	N3A—C9—N3—C10	−54.4 (10)
C1—C2—C3—O2	179.5 (3)	N2—C9—N3—C10	−157.7 (5)
O1—C2—C3—C4	−178.8 (3)	S1—C9—N3—C10	35.9 (8)
C1—C2—C3—C4	−0.9 (5)	C9—N3—C10—C11	−172.4 (6)
O2—C3—C4—C5	−180.0 (3)	N2—C9—N3A—C10A	139.6 (6)
C2—C3—C4—C5	0.5 (5)	N3—C9—N3A—C10A	51.5 (9)
C3—C4—C5—C6	−0.7 (5)	S1—C9—N3A—C10A	−54.8 (9)
C4—C5—C6—C1	1.3 (5)	C9—N3A—C10A—C11A	155.8 (9)
C2—C1—C6—C5	−1.7 (5)		

### Hydrogen-bond geometry (Å, º)

**Table:**

*D*—H···*A*	*D*—H	H···*A*	*D*···*A*	*D*—H···*A*
O1—H1···S1^i^	0.87 (4)	2.42 (4)	3.169 (3)	145 (3)
N2—H2···O1^i^	0.82 (3)	2.29 (4)	3.010 (4)	147 (3)

Symmetry code: (i) −*x*+1, −*y*+1, −*z*+1.
